# Comparative transcriptomics reveals the molecular genetic basis of pigmentation loss in *Sinocyclocheilus* cavefishes

**DOI:** 10.1002/ece3.7024

**Published:** 2020-11-19

**Authors:** Chunqing Li, Hongyu Chen, Yinchen Zhao, Shanyuan Chen, Heng Xiao

**Affiliations:** ^1^ Yunnan Key Laboratory for Plateau Mountain Ecology and Restoration of Degraded Environments School of Ecology and Environmental Sciences Yunnan University Kunming China; ^2^ School of Life Sciences Yunnan University Kunming China

**Keywords:** cavefish, comparative transcriptomics, pigmentation, *Sinocyclocheilus*, troglomorphic trait

## Abstract

Cave‐dwelling animals evolve distinct troglomorphic traits, such as loss of eyes, skin pigmentation, and augmentation of senses following long‐term adaptation to perpetual darkness. However, the molecular genetic mechanisms underlying these phenotypic variations remain unclear. In this study, we conducted comparative histology and comparative transcriptomics study of the skin of eight *Sinocyclocheilus* species (Cypriniformes: Cyprinidae) that included surface‐ and cave‐dwelling species. We analyzed four surface and four cavefish species by using next‐generation sequencing, and a total of 802,798,907 clean reads were generated and assembled into 505,495,009 transcripts, which contributed to 1,037,334 unigenes. Bioinformatic comparisons revealed 10,629 and 6,442 significantly differentially expressed unigenes between four different surface‐cave fish groups. Further, tens of differentially expressed genes (DEGs) potentially related to skin pigmentation were identified. Most of these DEGs (including *GNAQ*, *PKA*, *NRAS*, and *p38*) are downregulated in cavefish species. They are involved in key signaling pathways of pigment synthesis, such as the melanogenesis, Wnt, and MAPK pathways. This trend of downregulation was confirmed through qPCR experiments. This study will deepen our understanding of the formation of troglomorphic traits in cavefishes.

## Graphical abstract

We conducted a comparative histology and comparative transcriptomics study on the skin of eight Sinocyclocheilus species, including four surface and four cavefish species. The result suggests that these differentially expressed genes (DEGs) may be responsible for pigmentation loss in the Sinocyclocheilus cavefish species.

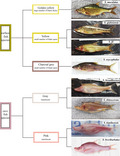

## 1 INTRODUCTION

Extreme habitats often foster the evolution of adaptive features (regressive evolution). Troglobites, a representative animal group that has adapted to harsh environments, exhibit a set of sensory, morphological, physiological, and behavioral traits that have arisen from long‐term adaptation to the perpetual darkness of the caves. The study on these traits will contribute not only to an improved understanding of the genetic basis of this evolutionary process but also help to reveal the processes of speciation and the function of individual genes. Loss of pigmentation, a characteristic trait of cave‐dwelling species, has received much attention in recent years. However, the regressive evolution mechanisms of reduction of skin pigmentation in cave animals need further investigation (Jeffery, 2009). Some cave‐dwelling creatures have been studied to decipher the link between the pigmentation and the underlying genetic mechanisms. For example, previous research on cave tetra revealed that the loss‐of‐function mutation of alleles of *oca2* (Oculocutaneous albinism II) leads to albinism in these fishes (Protas et al., 2006). Gross et al. discovered that the “brown” phenotype of *Astyanax mexicanus* may be responsible by the sequence mutations of the *MC1R* (melanocortin‐1 receptor) gene (Gross et al., 2009). Another study showed that albinism in cave‐dwelling planthoppers (*Oliarus polyphemus*) is caused by a defect in the conversion of L‐tyrosine to L‐DOPA in the melanin synthesis pathway (Bilandzija et al., 2012).

Color patterns of fish skins are related to the distribution and composition of pigment cells, including melanophores, erythrophores, xanthophores, iridophores, leucophores, and cyanophores, which are derived from neural crest cells (Parichy, 2006). The formation of fish skin pigmentation is orchestrated by multiple genes and regulatory factors at different genetic levels. More than 125 pigment genes (e.g., *TYR* [tyrosinase], *SILV* [premelanosome protein gene], *SOX10* [SRY‐box transcription factor 10], *MITF* [microphthalmia‐associated transcription factor], *MC1R*) have been identified to perform critical functions in pigmentation in many fish species (Parichy, 2006).

The wild freshwater teleost genus *Sinocyclocheilus* (Cypriniformes: Cyprinidae) is a treasured endemic fish genus, distributed in the karst regions of the east and northwest Yungui Plateau, Guangxi, southwestern China. This genus is composed of more than 55 know species (Meng et al., 2013). Owing to distinct differences in terms of phenotype and habitat, both morphotypes (cave and surface) exist within one genus. High species diversity and skin phenotypic variation make *Sinocyclocheilus* particularly suitable for studying the molecular genetic mechanisms underlying the evolution of differential pigmentation. Previous studies on three *Sinocyclocheilus* species (*Sinocyclocheilus graham* [surface fish], *Sinocyclocheilus rhinocerous* [cavefish], and *Sinocyclocheilus anshuiensis* [cavefish]) have shown that the expression of *oca2*, *Tyr*, *Tyrp1* (tyrosinase‐related protein1), and *Mpv17* (mitochondrial inner membrane protein 17) was lower in the skin of *Sinocyclocheilus* cavefish than that in the surface fish (Yang et al., 2016). However, considering the high species diversity and habitat differences, it is necessary to study more *Sinocyclocheilus* fish species to understand additional evolutionary mechanisms underlying pigment loss.

In this study, we conducted comparative histology and comparative transcriptomics analysis to examine phenotypic data and transcript profiles from the skins of four surface fishes (*Sinocyclocheilus* *maculatus*, *Sinocyclocheilus qiubeiensis*, *Sinocyclocheilus jii*, and *Sinocyclocheilus oxycephalus*) and four cavefish species (*Sinocyclocheilus tianlinensis*, *Sinocyclocheilus brevibarbatus*, *Sinocyclocheilus broadihornes*, and *S. rhinocerous*), all belonging to genus *Sinocyclocheilus*. The main goals of this study were to (a) obtain phenotypic data of different body colors at the histological level, and (b) obtain the skin transcriptome profiles of four *Sinocyclocheilus* cave‐dwelling and four *Sinocyclocheilus* surface fish species with Illumina sequencing technology, (c) select pigmentation‐related differentially expressed genes (DEGs) by comparing four cave‐surface fish groups based on skin transcriptome profiles, and, finally, (d) to identify and validate key candidate genes linked to the differential skin pigmentation between cave and surface fish species through pathway and function annotation of DEGs and qPCR. Our results are expected to provide novel insights into the transcriptional regulation of pigment‐related genes in adaptive evolution, leading to a better understanding of the molecular regulation mechanisms of troglomorphic traits in cavefishes.

## 2 MATERIALS AND METHODS

### 2.1 Sample collection and photograph with a digital camera

The eight *Sinocyclocheilus* species were captured in Yunnan Province and Guangxi Zhuang Autonomous Region, China, with three biological replicates for each species (Table 1). Pictures were taken of fresh fish immediately after capture. After general anesthesia with 30 mg/L of MS‐222 anesthetic (3‐aminobenzoic acid ethyl ester methanesulfonate; Sigma‐Aldrich), all lateral skin tissues were surgically excised, divided into two sections, collected into two sterile tubes for comparative histological and transcriptomics (tissue immersed into RNAlater) analysis, and placed in liquid nitrogen. After euthanizing the fish, other tissues (muscles) and organs (heart, liver, brain, etc.) were collected for other studies. The samples were stored at −80°C in the laboratory. Since the cavefish die soon after being removed from the cave habitat, to avoid RNA degradation, sterile instruments were prepared in advance, and all tissue sampling was performed in the field immediately after the fish were collected from the water. During the sampling process, all animal experiments were conducted with the approval of the local Ethical Committee at Yunnan University, in accordance with China's local and global ethical policies (Grant No: ynucae 20190056), and all procedures were approved and assisted by the local government.

**TABLE 1:** Sampling details of each *Sinocyclocheilus* species

Species		Location	Type	Sunlight intensity in Habitat	Gender	Age	Number
M	*Sinocyclocheilus maculatus*	N:23°4′; E:104°16′	Surface	High	All male	Adult	3
Q	*Sinocyclocheilus qiubeiensis*	N:24°05′; E:104°13′	Surface	High	All male	Adult	3
ji	*Sinocyclocheilus jii*	N:25°20′; E:110°9′	Surface	High	All male	Adult	3
J	*Sinocyclocheilus oxycephalus*	N:24°48′; E:103°18′	Surface	High	All male	Adult	3
T	*Sinocyclocheilus tianlinensis*	N:24°60′; E:106°30′	Cave	No sunlight	All male	Adult	3
D	*Sinocyclocheilus brevibarbatus*	N:24°10′; E:108°10′	Cave	No sunlight	All male	Adult	3
K	*Sinocyclocheilus broadihornes*	N:24°48′; E:103°18′	Cave	No sunlight	All male	Adult	3
X	*Sinocyclocheilus rhinocerous*	N:24°46′; E:104°17′	Cave	Weak	All male	Adult	3

For surface fish, we took fish images immediately after fishing. However, for cavefish, the sampling is done in deep caves (completely dark), and therefore, it cannot be photographed immediately (flash photography would produce chromatic aberrations). Thus, we temporarily placed the fish in a portable fishing case and supported with oxygen using a small aeration pump. Once outside the cave, the cavefish was sheltered from light the whole time to prevent skin pigment deterioration, except for taking photos under natural light.

### 2.2 Paraffin sections

The lateral skin samples from collected fishes were cut into 0.8 cm × 1.0 cm × 0.2 cm pieces and fixed with 10% neutral formaldehyde for 10‒24 hr, washed, and dehydrated through a graded series of ethanol (50%, 70%, 80%, 95%, and 100%). Paraffin embedding was carried out using a paraffin embedding station (Leica EG1140H; Leica Microsystems). The paraffin blocks were sliced into 6‐μm‐thick sections (Leica RM2016; Leica Microsystems), dried at 38°C for 7‒24 hr in the oven, and stained with haematoxylin–eosin. Sections were imaged using an optical microscope (OLYMPUS BX 51; Olympus).

### 2.3 RNA quantification and quality

RNA purity was checked using a NanoPhotometer® spectrophotometer (IMPLEN), and RNA concentration was measured using a Qubit^®^ RNA Assay Kit in a Qubit^®^2.0 Fluorometer (Life Technologies). RNA samples were run on 1% agarose gels, and their integrity was checked using an RNA Nano 6000 Assay Kit and the Agilent Bioanalyzer 2100 system (Agilent Technologies).

### 2.4 Library preparation for transcriptome sequencing

A total of 3 μg of RNA sample from each fish was used as input material. Sequencing libraries were generated using a NEBNext^®^Ultra™ RNA Library Prep Kit for Illumina® (New England BioLabs) following the manufacturer's recommendations, and index codes were added to attribute sequences to each sample. Briefly, mRNA was purified from total RNA using poly‐T oligo‐attached magnetic beads. Fragmentation was carried out using divalent cations at elevated temperature in NEB Next First Strand Synthesis Reaction Buffer (5×). The first‐strand synthesis of complementary DNA (cDNA) was completed using random hexamer primer and M‐MuLV Reverse Transcriptase (RNase H‐). Next, the synthesis of second‐strand cDNA was carried out using DNA Polymerase I and RNase H. Then, the synthesized cDNA was subjected to end repair and remaining overhangs were converted into blunt ends via polymerase treatment. After adenylation of the 3ʹ ends of DNA fragments, NEBNext Adaptor with a hairpin loop structure was ligated to prepare for hybridization. The size of target fragments selected for cDNA library was 150–200 bp in length. The fragments were purified with AMPure XP system (Beckman Coulter). Then, size‐selected, adaptor‐ligated cDNA was incubated at 37°C for 15 min, followed by 5 min at 95°C by using USER Enzyme (3 μl; New England BioLabs) before PCR. Finally, the library quality was assessed on the Agilent Bioanalyzer 2100 system after PCR using Phusion High‐Fidelity DNA polymerase, universal PCR primers, and index (X) primer (New England BioLabs) and product purification (AMPure XP system; Beckman Coulter).

### 2.5 Clustering and sequencing

Clone clusters were generated on Illumina cBot Cluster Generation System, using TruSeq PE Cluster Kit v3‐cBot‐HS (Illumina), according to the manufacturer's instructions. After cluster generation, high‐throughput sequencing of library preparations was performed on Illumina Hiseq 2000, and paired‐end reads were generated.

### 2.6 Data analysis

The raw reads were cleaned by removing reads containing adapter, reads containing poly‐N, and low‐quality reads through in‐house Perl scripts. Furthermore, to ensure the high quality of the data used for downstream analyses, the quality (Q20, Q30, GC‐content, and sequence duplication level of the clean data) of the clean data was determined. Clean and high‐quality transcriptome from the eight fish species were assembled using Trinity (min_kmer_cov: 2; other parameters: default values) (Grabherr et al., 2011). The whole sequence data were submitted to the DRYAD database (https://datadryad.org/stash/share/t5cZIXoVUgyhpzEP6z-GN6xjc5EU3TvPPEwbdIo7siI).

### 2.7 Gene function annotation

Gene functions of unigenes were annotated based on the homology searches of the following major public databases: NCBI nonredundant protein (nr), protein families (Pfam), euKaryotic Orthologous Groups (KOG)/Clusters of Orthologous Groups of proteins (COG)/nonsupervised orthologous groups (eggNOG), Swiss‐Prot (a manually annotated and reviewed protein sequence database), Kyoto Encyclopedia of Genes and Genomes (KEGG), and Gene Ontology (GO).

### 2.8 Differential expression analysis

According to different pairing modes, we investigated more than 30 combinations of pair‐group among the eight species, and four species pairs (M vs. K, Q vs. X, ji vs. T, J vs. D; Table 1) were randomly selected for further description. The target genes were identified according to the following three criteria: (a) genes with expression log_2_^|Foldchange|^ ≥ 2 and false discovery rates (FDR) ≤0.001 were considered to be differentially expressed. (b) The functions and pathways of these DEGs were involved in the formation of fish skin pigmentation. (c) The regulation trends (upregulation or downregulation) of these DEGs must be consistent across at least three of the four groups. Differential expression analysis of all four groups was performed using the DESeq R package (1.10.1) (Love et al., 2014). DESeq analyses count data from high‐throughput sequencing to determine differential expression using a model based on a negative binomial distribution. The resulting P‐values were adjusted using the Benjamini–Hochberg procedure to factor in FDR. DEGs were determined by comparing levels of gene expression within each group after using Bowtie and RSEM to calculate fragments per kilobase of exon per million mapped reads (FPKM) (Langmead et al., 2009; Li & Dewey, 2011). Genes with expression log_2_^|Foldchange|^ ≥ 2 and FDR ≤ 0.001 were considered to be differentially expressed.

### 2.9 DEG functional annotation

GO enrichment analysis of DEGs was implemented by the topGO R packages (Alexa & Rahnenfuhrer, 2006), using a Kolmogorov–Smirnov test. KEGG (Kanehisa et al., 2004) is a database for understanding high‐level functions and utilities of biological systems, such as the cell, organism, and ecosystem, from molecular‐level information, especially large‐scale molecular datasets generated by genome sequencing and other high‐throughput experimental technologies (http://www.genome.jp/kegg/). We used KOBAS (Xie et al., 2011) software to test the statistical enrichment of DEGs in KEGG pathways.

### 2.10 Validation of DEGs by qPCR

RNA‐seq results of a total of 9 DEGs were validated by qPCR; β‐actin (housekeeping gene) was used as an internal reference. qPCR analysis using the same RNA samples as for the transcriptome profiling was performed using qTOWER2.2 (Analytik Jena, Jena, Germany); the primer sequences are listed in (Table S1). The amplification conditions were as follows: initial denaturation at 95°C for 3 min, followed by 39 cycles of denaturation at 95°C for 10 s and extension at 58°C for 30 s. Finally, melting curves were generating from 60 to 95°C. All reactions were performed with three technical and three biological replicates. Relative gene expression was calculated with the 2^−ΔΔCT^ method using qPCRsoft 3.2 software (The value of threshold cycle Ct was used to calculate the DEG expression fold change. ΔCt = Ct_target genes_ − Ct_β‐actin_, ΔΔCt = ΔCt_control_ − ΔCt_Indicated condition,_ log_2_^(FC)^ = 2^−(ΔΔCt)^.

### 2.11 Weighted gene coexpression network analysis (WGCNA)

We used the R package WGCNA for weighted correlation network analysis. By using gene expression data from the skin tissue of cave and surface fish, we constructed a coexpression network to find additional important genes associated with each skin color phenotype. First, we constructed a gene–gene similarity network (Pearson's correlation) for all unigenes. In this analysis, we filtered unigenes with expression quantity <1 to calculate the optimal power value, and transformed these values into an adjacency matrix under the soft power (beta = 7). Next, all unigenes were hierarchically clustered, and the network was divided into modules. Gene modules corresponding to the branches cutoff of the gene tree were color‐coded (networkType: signed; soft power: 7; minModuleSize: 50; minKMEtoStay: 0.3; mergeCutHeight: 0.20). The “gray” module contained unigenes that could not be associated with any expression patterns. To find the core genes (hub genes) that are significantly associated with the phenotypic trait, we focused on the most highly correlated modules (*R* ^2^>|.5|, *p* < .005) and the top three most correlated genes were selected for further analysis.

## 3 RESULTS

### 3.1 Color observations in *Sinocyclocheilus* skin

**FIGURE 1:**
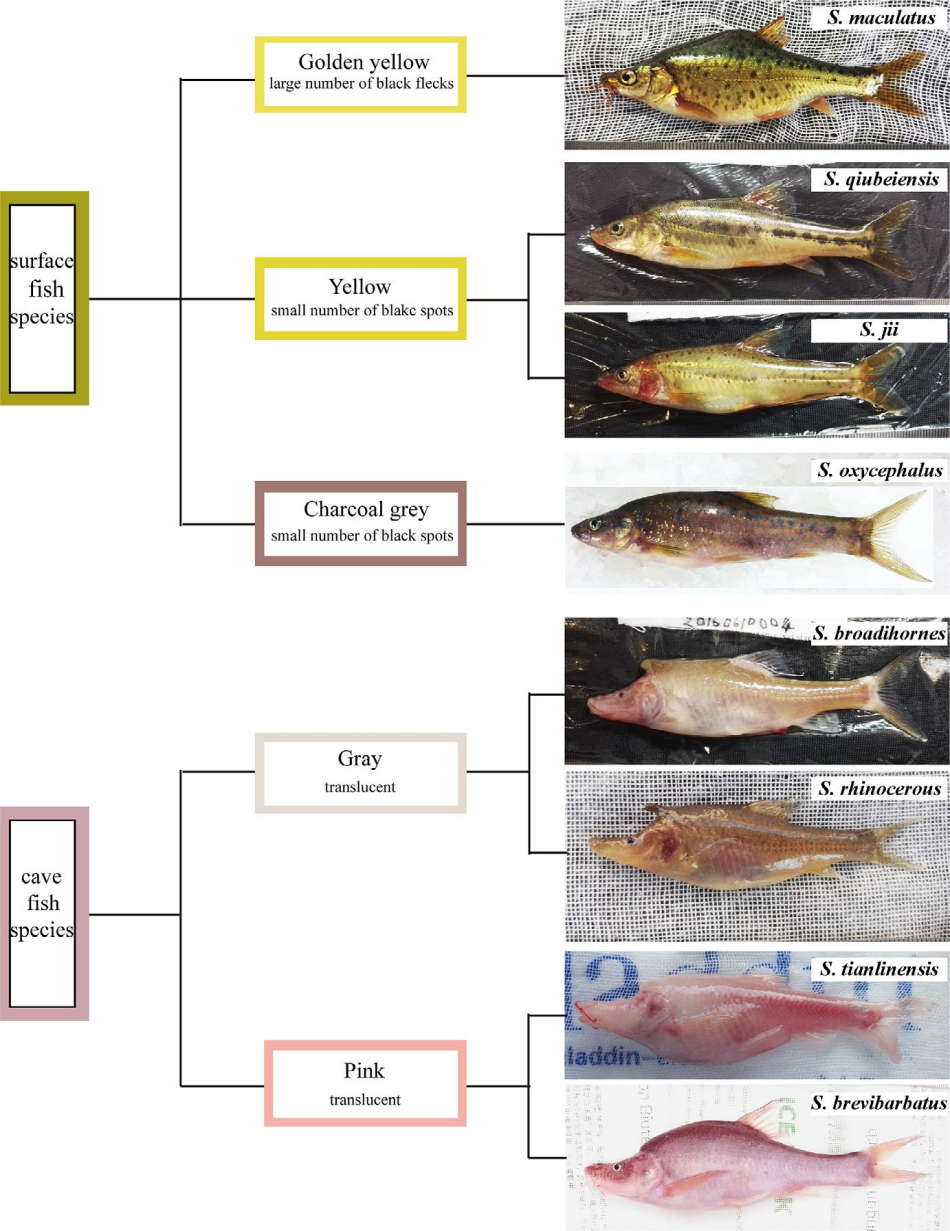
Digital photographs of eight *Sinocyclocheilus* species in living state

The eight *Sinocyclocheilus* species were photographed in the living state with a digital camera (Figure 1), and skin color was noted. Based on the body color, the four surface fish species were divided into three different subtypes within the same type of habitat. *Sinocyclocheilus maculatus* skin has a large number of black flecks and appears golden yellow, whereas the skins of *S. qiubeiensis* and *S. oxycephalus* exhibit yellow coloration but with fewer flecks. Distinct from the other three surface fish species, *S. jii* has a charcoal gray body color with little black spots. The four cave‐dwelling *Sinocyclocheilus* species were divided into two color subtypes: *S. broadihornes* and *S. rhinocerous* belong to the gray translucent body color type, and *S. tianlinensis* and *S. brevibarbatus* belong to the pink translucent body color type.

**FIGURE 2:**
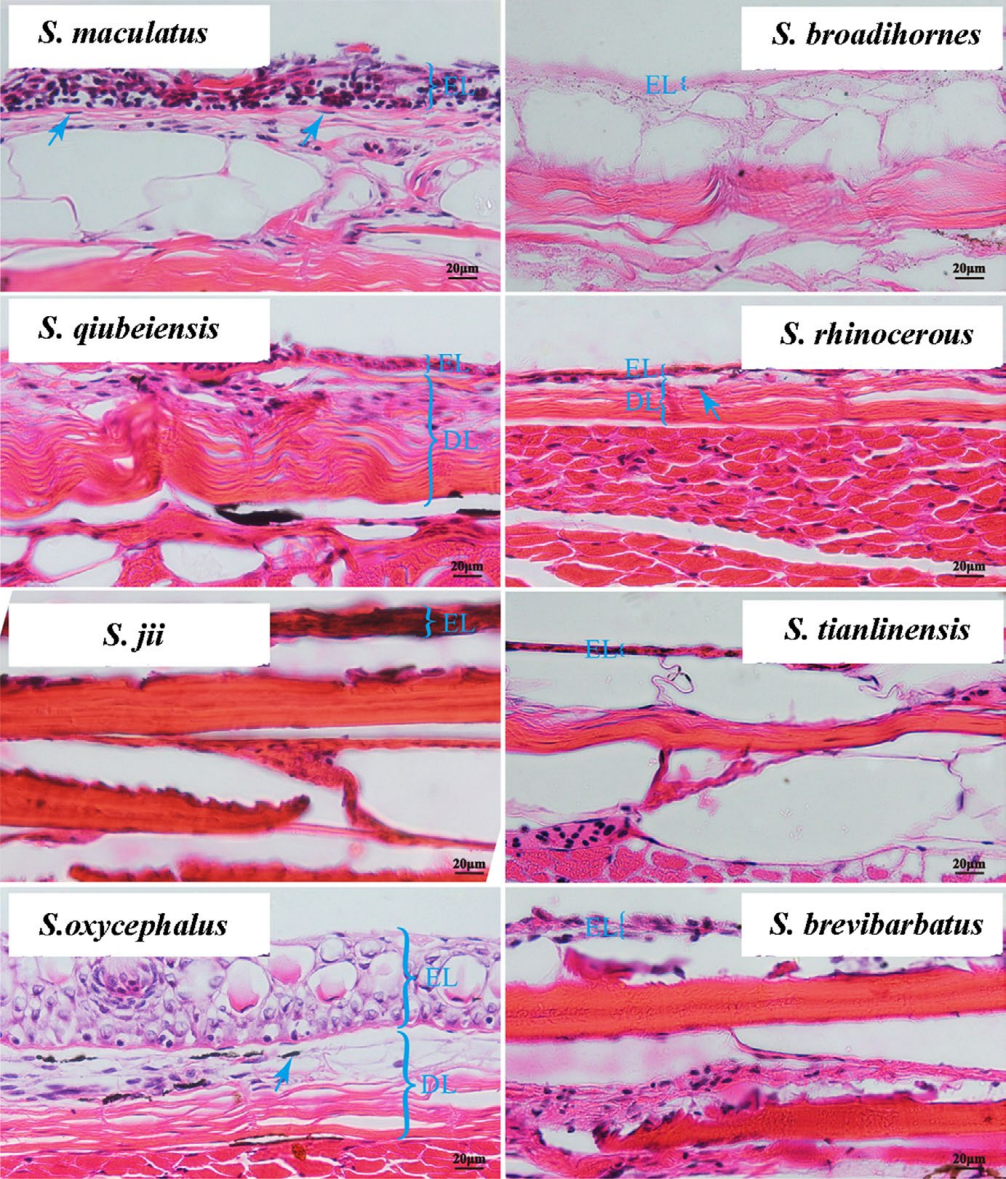
The stereoscopic microscopic photographs of skin paraffin sections of eight *Sinocyclocheilus* species. Melanophores are indicated by blue arrows (magnification 40×). EL, epidermal layer; DL, dermal layer. Melanocytes were indicated with blue arrow

The photographs taken of the same area on the lateral skin of the eight species using a stereoscopic microscope showed the presence of black dots composed of melanocytes in all surface fishes. However, the melanocytes were not as evident in the cavefish. For the four surface fish species, the difference in the light used for illumination at the time of taking the photograph may explain the differences in melanocyte shape. The star‐like shape of melanocytes of *S. qiubeiensis* and *S. jii* may be due to the expansion of intracellular melanosomes in melanocytes under low light conditions. Interestingly, the melanocytes in cavefish differed significantly in the distribution patterns, melanocytes abundance, and intracellular arrangement of melanosomes. The melanocytes in *S. broadihornes* and *S. brevibarbatus* skins were star‐shaped, and those in *S. rhinocerous* and *S. tianlinensis* had dendritic shape. Furthermore, the fine‐lined melanocytes in *S. tianlinensis* skin were the faintest among the four cavefish species observed. The melanocytes of the four surface fish species were thicker and darker than those of the cavefish species, and fewer melanin granules were observed in the cavefish skin (Figure S1). The detailed distribution and composition patterns of melanocytes are shown in Figure 2.

### 3.2 Sequencing and de novo assembly

Sequencing of the skin transcriptomes of the eight *Sinocyclocheilus* species using the Illumina Hiseq 2000 platform produced 802,798,907 clean reads and 237,946,156,516 bases from the constructed sequencing libraries. The read and base numbers, GC‐content, and other parameters are presented in Table S2. High‐quality reads were de novo assembled using Trinity, generating 505,495,009 transcripts. All transcripts contributed to 1,037,334 unigenes with an average length of 820.24 bp and N50 of 1,379 bp. Information on sequence length distribution and other details of transcripts and unigenes are shown in Table S3.

### 3.3 Unigene functional annotation

**FIGURE 3:**
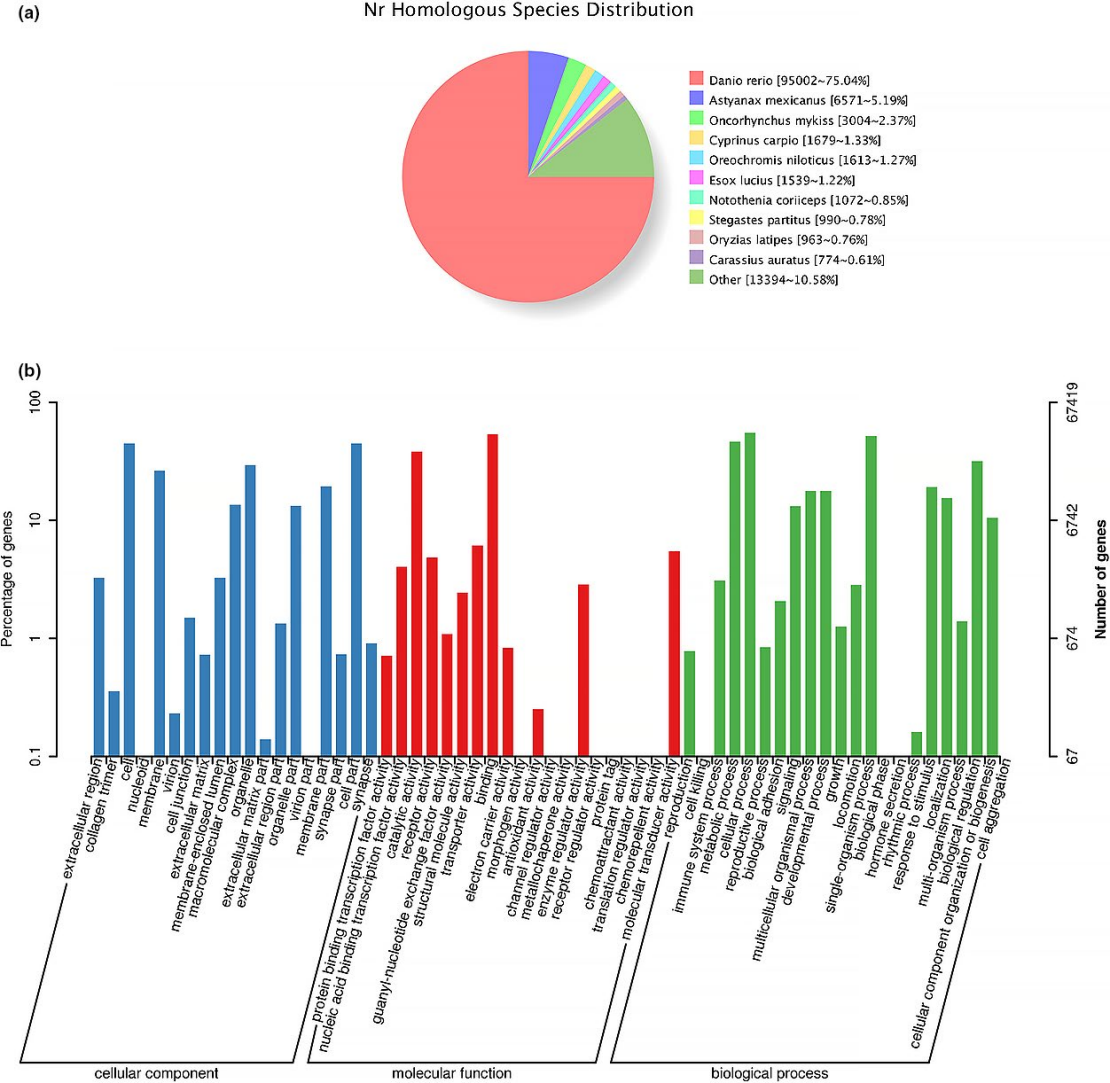
Annotation of unigenes form the eight species of *Sinocyclocheilus*, according to Nr and GO. (a) Similarity analysis of the unigenes against nonredundant Nr database; (b) Gene Ontology (GO) classification of unigenes for skin transcriptome of eight *Sinocyclocheilus*

A total of 131,766 unigenes from the eight species of *Sinocyclocheilus* were annotated using several databases (including Nr, GO, COG, and KOG, Table S4). The BLASTx similarity analysis of the unigenes against the NCBI Nr database found matches for most of the unigenes (126,654) in the Nr database. Most of the unigenes had the highest homology with protein sequences of seven fish species (Figure 3a): *Danio rerio* (75.04%), *A. mexicanus* (5.19%), *Oncorhynchus mykiss* (2.37%), *Cyprinus carpio* (1.33%), *Oreochromis niloticus* (1.27%), *Esox lucius* (1.22%), and *Notothenia coriiceps* (0.85%). To further annotate the functions of unigenes, three databases (COG, eggNOG, and KOG) were aligned to unigenes. The results of the functional classification of unigenes were as follows. In all three databases, the cluster of general function prediction (R) had the largest proportion. The proportion of signal transduction mechanisms (T) was second in the NOG and KOG databases, whereas, in the COG database, only a few unigenes were annotated in this functional class. In addition, the proportion of some clusters showed a consistent trend across the three databases in cell motility (N), nuclear structures (Y) and cell wall/membrane/envelope biogenesis (M), posttranslational modification, protein turnover, and chaperones (O), and transcription (K). However, the proportions of most categories in these three databases were different, especially in S (function unknown) and L (replication, recombination, and biogenesis; Figure S2). Further, GO terms were assigned to 67,419 unigenes using GO database annotation. The most abundant GO terms in the GO classes were binding (GO: 0005488) and catalytic activity (GO: 0003824) in MF (molecular function); cell (GO: 0005623) and cell part (GO: 0044464) in CC (cellular component); and cellular process (GO: 0009987) and metabolic process (GO: 0008152) in BP (biological process; Figure 3b). To further identify the biological pathways of these unigenes, they were mapped to the KEGG database. A total of 60,928 unigenes were mapped to 285 known KEGG pathways. The top five KEGG pathways with the highest number of unigenes were the MAPK signaling pathway (Ko04010), endocytosis (Ko04144), focal adhesion (Ko04510), regulation of actin cytoskeleton (Ko04810), and calcium signaling pathway (Ko04020). Furthermore, pigment‐related pathways such as melanogenesis (Ko04916) and the Wnt signaling pathway (Ko04310) were included in the top 30 pathways (Figure S3).

### 3.4 DEG analysis

DEGs were identified in the four fish groups (M vs. K, Q vs. X, ji vs. T, J vs. D).

**FIGURE 4:**
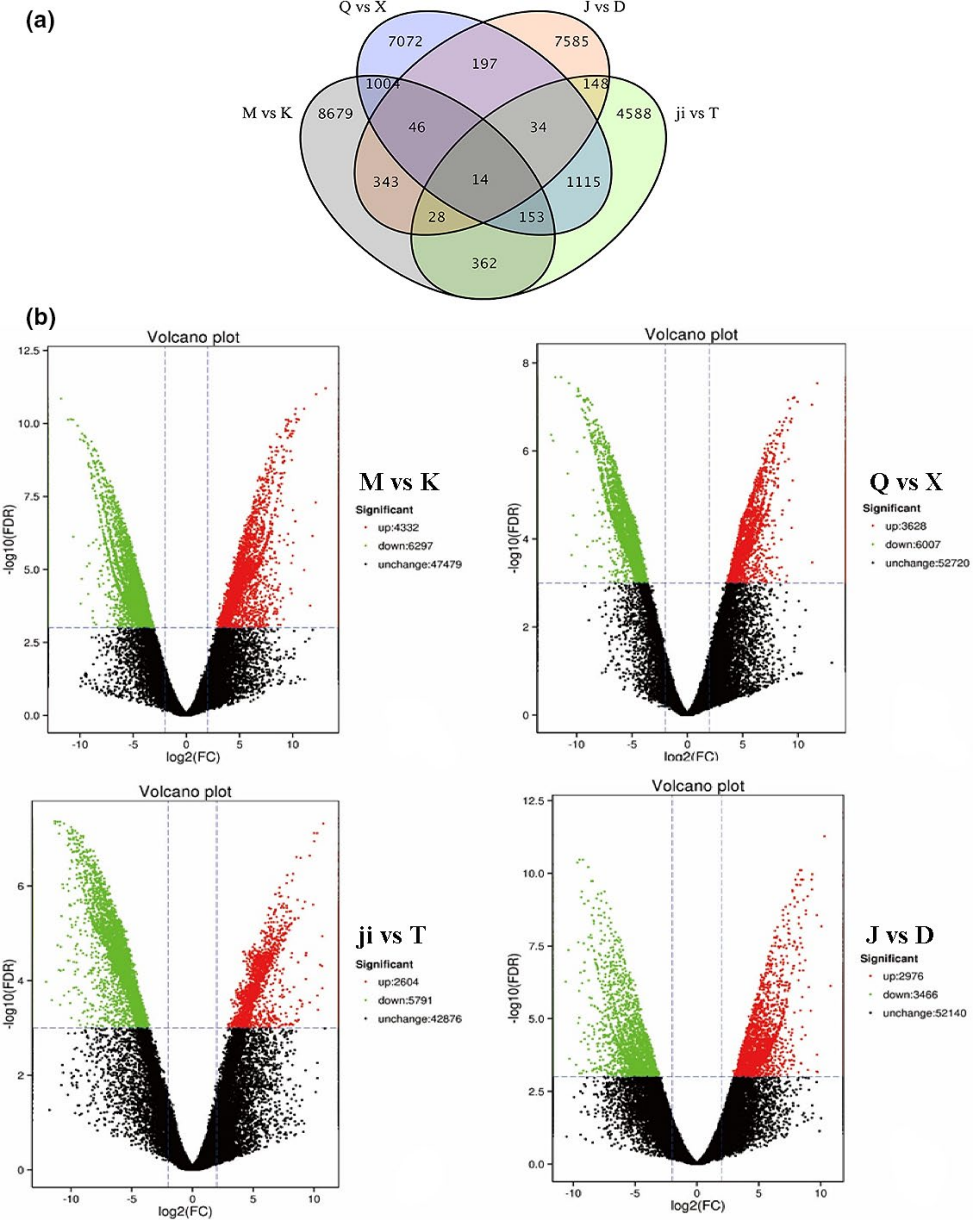
(a) Venn diagram displaying the number of differentially expressed genes (DEGs) unique to or shared between each surface‐cave group Numbers in each section of the figure show the number of DEGs (log_2_|Foldchange| ≥ 2 and false discovery rates (FDR) of ≤0.001). (b) Number of differentially expressed genes of four surface‐cave fish groups. *X*‐axis shows the differences in expression given as the log values, and the *Y*‐axis shows significant differences in expression as negative log values in the Volcano plot; upregulated genes and downregulated genes are indicated by red dots and green dots, respectively. Nondifferentially expressed genes are indicated by black dots. (a) M vs. K; (b) Q vs. X; (c) Ji vs. T; (d) J vs. D

We obtained a total of 35,101 DEGs from the four fish groups. M vs. K group had the highest number of DEGs (10,629 DEGs: 4,332 upregulated and 6,297 downregulated) followed by Q vs. X (9,635 DEGs: 3,628 upregulated and 6,007 downregulated) (Figure 4b). In every surface fish and cavefish group compared, the proportion of downregulated genes was higher than that of upregulated genes (Table S5). As shown in the Venn diagram, Q vs. X and ji vs. T had the most shared DEGs (their habitats are not more similar than other groups) The more details of unique and shared DEGs between each surface‐cave group are displayed in Figure 4a.

### 3.5 DEG functional and pathway analysis in the databases

GO, KEGG, and COG annotations were performed to identify the functions and biochemical pathways of DEGs to further filter the candidate genes that may be responsible for differences in skin pigment (Table S5). DEGs in all four groups were classified into three functional categories (biological process, cellular component, and molecular function) in the GO database. Most of the DEGs of the four groups were annotated to seven GO subcategories, namely “cellular process,” “single‐organism process,” “metabolic process,” “cell,” “cell part,” “binding,” and “catalytic activity.” However, partial subcategories were only annotated in some groups; for example, the subcategory “channel regulator” was annotated only in groups M vs. K and Q vs. X, and “extracellular matrix part” was found only in groups ji vs. T and J vs. D. (Figure S4). GO enrichment analysis of DEGs with Kolmogorov–Smirnov tests revealed that six GO subcategories, homophilic cell adhesion via plasma membrane adhesion molecules, ionotropic glutamate receptor signaling pathway, extracellular region, structural constituent of ribosome, ionotropic glutamate receptor activity, and extracellular‐glutamate‐gated ion channel activity were significantly enriched (*p* < .001) in at least three of the four groups (Table S6).

To further study the function and BPs of DEGs, and identify target genes annotation of DEGs (four groups) was performed using KEGG pathways (Figure S5). Under the category of the biochemical pathway in the KEGG classification, DEGs in all four comparison groups were associated with cellular processes, environmental information processing, genetic information processing, human diseases, metabolism, and organismal systems. Furthermore, the KEGG subcategories with a high proportion of DEGs in the four groups were endocytosis, protein processing in the endoplasmic reticulum, ribosome, and MAPK signaling pathway. Interestingly, some pathways showed differences among the four groups, such as the cardiac muscle contraction pathway, which was annotated in all groups except for the Q vs. X group, and the RIG‐l‐like receptor signaling pathway and the fatty acid metabolism, which were only observed in the ji vs. T and M vs. K groups, respectively. In this study, DEGs in all four groups were partially annotated to some pigment‐related pathways such as the melanogenesis, MAPK signaling, and Wnt signaling pathways.

### 3.6 Target DEGs selection and expression trend validation

After the functional analysis and pathway annotation of DEGs in the four groups, we selected target genes according to the restrictions stated above. Five DEGs were found in the melanogenesis pathway, and the expression of these genes in all surface‐cave pairs was checked. Furthermore, seven DEGs involved in Wnt signaling, MAPK signaling, and apoptosis pathways, which are putatively involved in skin pigmentation, were selected from a total of 35,101 DEGs. Except for *SFRP2*, most of the DEGs were downregulated in *Sinocyclocheilus* cavefishes (Table 2).

**TABLE 2:** Differentially expressed genes (DEGs) related to pigmentation in each surface‐cave group

Pathway	Gene name	FDR	MvsK	QvsX	jivsT	JvsD	MvsT	MvsD	MvsX	QvsT	QvsD	QvsK	jivsD	jivsK	jivsX	JvsT	JvsK	JvsX
Melanogenesis			log_2_^|FC|^				log_2_^|FC|^											
	*GNAQ*	<0.001	−6.14	−4.76	−4.59	−4.41	<|2.0|	−Inf	−6.10	−2.62	−8.02	<|2.0|	−5.34	−3.66	−5.14	−5.86	−2.13	<|2.0|
	*AC*	<0.001	−5.04	−4.46	−4.26	−Inf	−3.30	−Inf	<|2.0|	<|2.0|	−Inf	1.37	−Inf	−3.00	−3.31	<|2.0|	−2.55	<|2.0|
	*NRAS*	<0.001	−Inf	−5.53	−3.49	−4.80	−2.24	−3.90	−Inf	−2.94	−Inf	−Inf	−5.39	−Inf	−Inf	−3.03	−Inf	−2.45
	*SFRP2*	<0.001	−5.91	3.96	6.17	8.51	<|2.0|	3.20	<|2.0|	5.61	4.56	5.13	<|2.0|	<|2.0|	3.24	3.18	3.83	2.84
	*PKA*	<0.001	−5.73	−6.33	<|2.0|	−3.68	<|2.0|	−2.14	<|2.0|	<|2.0|	−5.24	−2.16	−6.20	−4.55	−5.68	−3.39	<|2.0|	−2.35

Pathway	Gene name	FDR	M versus K	Q versus X	ji versus T	J versus D
			log_2_^|FC|^			
Wnt signaling pathway	*DAAM1*	<0.001	−5.38	−4.81	−6.10	−2.58
MAPK signaling pathway	*TAO*	<0.001	−5.57	−8.14	−7.24	−5.39
	*P38*	<0.001	−6.54	−5.00	−5.47	−3.63
Apoptosis	*BID*	<0.001	−5.27	−5.5	−4.58	−Inf
	*BAD*	<0.001	−4.76	−4.78	−4.45	−Inf
	*BAX*	<0.001	−3.27	−Inf	−4.41	−4.30

Note

“Inf” and “−Inf” indicate that the gene is not expressed in the cavefish or surface fish. “<|2.0|” indicate that the log_2_^|FC|^<2 (no significant difference)

**FIGURE 5:**
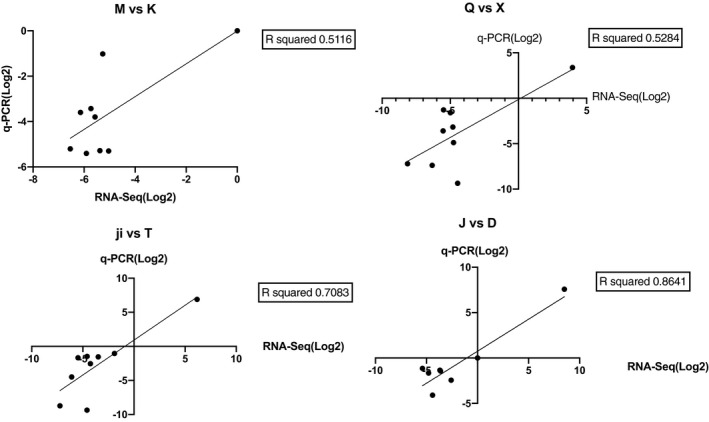
Quantitative real‐time PCR (qRT‐PCR) validation of differentially expressed genes (DEGs) in each surface‐cave group. The expression profile of nine genes was analyzed by qRT‐PCR using the same RNA as utilized for RNA‐Seq. Each average RNA‐Seq expression value was plotted against the corresponding qRT‐PCR value and fitted into a linear regression (conducted by Prism 8.4.0.)

The expression of a total of 9 selected DEGs was verified by qPCR to confirm the accuracy of the RNA‐Seq data (Figure 5).

### 3.7 Weighted gene coexpression network analysis (WGCNA)

**FIGURE 6:**
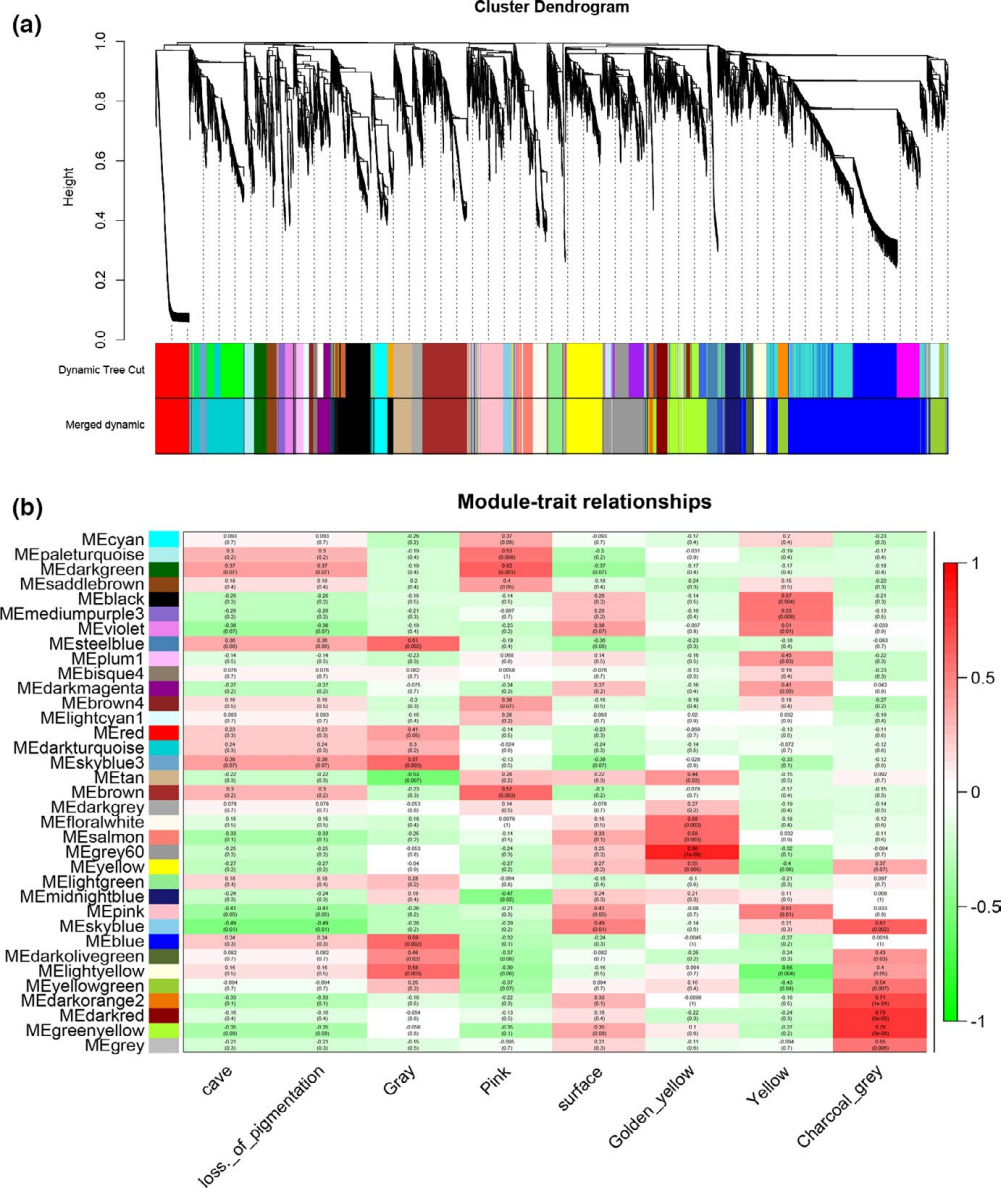
Coexpression modules conducted by WGCNA. (a) hierarchical cluster tree constructed by WGCNA shows coexpression modules (the gray module represents genes that are not assigned to specific modules). Each branch in the tree point connects to a gene. Genes were assigned to network modules by dynamic tree cut after the dynamic tree cut algorithm has been used to identify all modules. minModuleSize:30 (lowest number of genes in each module) and Merged dynamics (the modules with similar expression patterns (80%) were then merged.); (b) Trait–module associated heat map. Column: traits; row: modules. The number in each grid represents the correlations between the module and corresponding traits (correlation coefficients) and *p*‐value (in parentheses). Color of the grid indicates the correlation (the deeper the color, the stronger the correlation)

After filtering of genes with low levels of expression, the modules were divided based on clustering genes with similar expression patterns and calculating the correlation between the eigenvalue and traits of each module. The coexpression network was used to link gene expression to five skin color phenotypes quantitatively. As a result, all analyzed genes resulted in a total of 35 color modules. We found that the color subtypes of each cavefish and surface fish have different highly correlated modules (Figure 6). Here, we focused on the five most correlated modules: “steel blue,” “dark green,” “grey60,” “green‐yellow,” and “black” (*R* ^2^ > |.5|; *p* < .005) to determine hub genes. The top three hub genes based on eigengene connectivity in each module may play an important role in each trait. The details and functions of these genes are presented in Table 3.

**TABLE 3:** TOP 3 Hub genes identified by WGCNA in each skin color traits

Skin Color‐module	Unigene id	Gene	Gene function
gray‐steel blue (Cavefish)	BMK_Unigene_053214	C1q‐like	Inhibit the apoptosis et al
	BMK_Unigene_030559	fndc5	Fulfill manifold functions in tissue development and regulation of cellular metabolism.
	BMK_Unigene_030561	FN1	Fibronectins are involved in cell adhesion, cell motility, opsonization, wound healing, and maintenance of cell shape.
pink‐dark green (Cavefish)	BMK_Unigene_033481	PLEC	Cytoskeleton‐associated protein which links the keratin intermediate filaments to the transmembrane proteins of the hemidesmosomes.
	BMK_Unigene_276989	Tpbgl	Trophoblast glycoprotein‐like
	BMK_Unigene_160469	CRLF1	In complex with CLCF1, forms a heterodimeric neurotropic cytokine that plays a crucial role during neuronal development
Golden‐grey60 (Surface fish)	BMK_Unigene_176217	H1GD1A	Subunit of cytochrome c oxidase
	BMK_Unigene_181645	TMP3	Binds to actin filaments in muscle and nonmuscle cells
	BMK_Unigene_213257	CALML6	Calmodulin‐like protein 6 isoform X1
Yellow‐black (Surface fish)	BMK_Unigene_135409	FAM13B	GTPase activator activity
	BMK_Unigene_143803	USP2	Hydrolase that deubiquitinates polyubiquitinated target proteins such as MDM2, MDM4 and CCND1
	BMK_Unigene_130832	ATP5PF	Mitochondrial membrane ATP synthase
Grey‐green‐yellow (Surface fish)	BMK_Unigene_029491	PUP2	Proteasome subunit alpha type‐5
	BMK_Unigene_084262	lmnb2	Lamin B2
	BMK_Unigene_099265	SNRPG	Small nuclear ribonucleoprotein G

## 4 DISCUSSION

For cave‐dwelling fish species, the loss of evolutionarily undesirable traits is pivotal to control energy cost and may be one of the reasons for the convergent evolution of regressive traits; however, the regulatory mechanism underlying this process is complex (Protas et al., 2007). Studies on skin pigments in *A. mexicanus* have reported that different genes determine the degrees of skin pigment loss (brown and albino) (Gross et al., 2009; Protas et al., 2006). Besides, the mutations in these genes may be pleiotropic. Helena et al. found that the albinism of *A. mexicanus* caused by loss‐of‐function mutations in the oca2 gene is a by‐product of catecholamine‐related behaviors (anesthesia resistance), which is related to the capacity to be responsive to stimuli in the caves (reduced sleep) (Helena et al., 2018), which indicates that the cause of reduced pigmentation of cavefish may be more complicated than earlier believed, and there may be additional, yet unidentified pigment‐related genes.

The link between loss of pigmentation and the underlying genetic mechanisms in cave‐dwelling fishes can be better understood by investigating the pattern of transcriptional regulation of genes that may cause loss of skin pigmentation in *Sinocyclocheilus* cavefish. In this study, we selected eight representative (skin color and habitat type) *Sinocyclocheilus* species, which included four surface species (with functional eyes and pigmentation) and four cave species (with reduced or absent eyes and pigmentation) to evaluate the phenotypic differences in skin pigmentation between the two ecological types of *Sinocyclocheilus* species. We aimed at identifying and confirming key candidate genes linked to the diverse skin pigmentation between cave and surface fish species through comparative transcriptomics analysis.

### 4.1 Relationship between skin color and habitat of *Sinocyclocheilus* species

Skin color can be influenced by environmental interactions (Leclercq et al., 2010). During the long‐term sampling process and looking at lots of other of species within this genus, we found that the skin color of *Sinocyclocheilus* species varies widely among species of the surface also cave‐dwelling fish, but remains consistent within populations. The eight representative *Sinocyclocheilus* species selected in this study covered almost all the color types we have observed in the wild. Our comparative histological analysis of the two ecotypes of *Sinocyclocheilus* species clearly indicated the differences in distribution patterns, quantity, and melanosome intracellular arrays of melanocytes in the skin between cavefish species and surface fish species, suggesting that different habitats may be one of the crucial reasons for this difference. Melanocytes synthesize melanin to protect skin by absorbing ultraviolet radiation (UV) (Kim et al., 2018). Previous studies on fish, such as whitefish larvae (*Coregonus lavaretus*) (Winberg, 2000), red sea bream (*Pagrosomus major*) (Kumai, 2005), and rainbow trout (*O. mykiss*; Little, 1995) showed that the concentration of skin melanin and eumelanin precursor increase in response to UV exposure to adapt to the changing environment. Melanin can absorb specific wavelengths of light to protect the skin against UV radiation, resulting in the dark gray skin of zebrafish (Kelsh, 2004). However, troglobites live in completely enclosed underground habitats characterized by permanent darkness, and complete lack of autochthonous photosynthesis, leading to limited food resources (Bussotti et al., 2018). When adapting to these extreme environments, the accumulation of melanin in cavefish skin is a waste of limited resources (Culver & Pipan, 2009). Interestingly, among the four cavefish species, digital photography showed that the skin color of *S. rhinocerous* was the darkest but still transparent, and the stereoscopic microscopic photographs and paraffin section also confirmed that the number of melanocytes in the *S. rhinocerous* was greater than that in the other cavefish species. Li et al. (2000) studied the cave habitats at sampling sites of *S. rhinocerous* and found that this species occupies funnel‐shaped doline caves, which partially receive solar radiation; in contrast, other cavefishes, such as *Sinocyclocheilus altishoulderus*, live in completely enclosed underground rivers. This half‐open environment may partially explain its unique skin color.

Based on comparative histology, we divided the *Sinocyclocheilus* cavefish and surface fish body colors into two and three subtypes, respectively. The coexpression network of WGCNA was used to link gene expression with five skin color phenotypes quantitatively. The “steel blue” module and the “dark green” module were positively correlated with the cavefish gray and pink color traits, respectively, indicating that the two modules might play an important role in cavefish pigmentation. We found hub genes among the top three hub genes, *C1q‐like* (complement C1q) and *FN1* (fibronectin 1), in the “gray‐steel blue” module whose expression was related to solar ultraviolet radiation (UVR). Mei et al. found that C1q‐like is involved in UV‐induced apoptosis in zebrafish. After the UV exposure, the transcripts of C1q‐like were upregulated 3–4 fold (Mei et al., 2008). Fibronectin is a globular glycoprotein ubiquitous in the dermal extracellular matrix (ECM), and exposure to UV irradiation modulates *FN1* expression, thereby enhancing ECM degradation (Hibbert et al., 2017; Parkinson et al., 2015). Furthermore, in the “pink‐dark green” module, *PLEC* (plectin) is considered the top hub gene. Previous studies of human skin color have shown that the attenuated expression of *PLEC* leads to increased melanosome uptake by keratinocytes and skin hyperpigmentation after UVA exposure (Coelho et al., 2015). This finding may reconfirm that the differences in body color between cavefish in this study are related to the UV intensity among different habitats.

Wild *Sinocyclocheilus* species establish complex interactions with their habitats, which may require the development of different mechanisms or regulatory genes for each skin color subtype of cavefish such as in *S. rhinocerous* and *S. altishoulderus*. Furthermore, some surface fish or cavefish may occasionally change their habitat through the underground river for predation (Chen et al., 2019), which may be part of the reason for the diversity of body colors between the same type of fish. Complex wild habitats may have diverse influencing factors such as seasonal changes and diet, which may affect the formation of host skin pigmentation. Nevertheless, further research is needed to reveal the exact mechanisms underlying these variations.

### 4.2 DEGs related to loss of pigmentation of *Sinocyclocheilus* fish

The distribution of the genus *Sinocyclocheilus* is very narrow, and the habitat of each species is merely a single cave or one surface waterbody (Lunghi et al., 2019). Furthermore, a large number of variables (age, geographical location, and species) may affect gene transcription. As such, in the grouping, these surface‐cave fish pairs were combined only based on the “habitat type” to find the DEGs of each group, before comparisons were made. We believe that the candidate genes selected in this way could truly reflect the relationship between skin pigmentation and the two habitat types (cave and surface waterbody), rather than the effect of other variables.

Through comparative histology studies, we found that surface fish have a large number of melanocytes on the skin compared to cavefish. Previous researches have shown that two types of melanin (eumelanin and pheomelanin) are produced (Jiang et al., 2014) and stockpiled by the melanosomes in melanophores (Slominski et al., 2004). Previous studies have shown that the expression of melanin is regulated by multiple genes in the melanogenesis pathway, such as *Mc1r*, *MITF*, *GNAQ* (α melanocyte‐stimulating hormone). Accordingly, in this study, we first focused on the melanogenesis pathway for candidate DEGs screening and found five genes: *GNAQ*, *AC* (adenylate cyclase), *NRAS* (NRAS proto‐oncogene, GTPase), *SFRP2* (secreted frizzled‐related protein 2), and *PKA* (protein kinase) with significant differential expression, and further verified the expression trends of these five genes in all surface‐cave pairs.

In the melanogenesis pathway, *Mc1r* on the cell membrane is activated by α*‐MSH*, which results in the stimulation of Gnαq, thereby activating *AC* to increase the production of cAMP and *PKA* activation. *PKA* upregulates the expression of *MITF*, leading to a rise in melanin synthesis (Buscà & Ballotti, 2000; García‐Borrón et al., 2005; Oscar & Van, 2016; Van Raamsdonk et al., 2009), eventually leading to darker skin coloration through the intracellular dispersal of membrane‐bound pigment granules (melanosomes) within the melanophore (García‐Borrón et al., 2005; Hoekstra, 2006; Lin & Fisher, 2007; Yamaguchi et al., 2007). Notably, we found three genes, *GNAQ*, *AC*, and *PKA*, that showed a significant downregulation trend in most cavefish skins.

*GNAQ* encodes Gnαq, a subunit of a trimeric G protein complex that binds to the endothelin B receptor in melanocytes (Raamsdonk et al., 2004), and plays an important role in the regulation of pigment formation (Van Raamsdonk et al., 2009). We found that *GNAQ* was significantly downregulated in more than 75% of surface‐cave pairs (12/16 pairs). Recent research found that the expression of *GNAQ* in black mouse skin was significantly higher than that in the white mouse skin (Yin et al., 2015). In fish species, *GNAQ* showed a significant association with skin pigmentation in three spine sticklebacks (*Gasterosteus aculeatus*; Greenwood et al., 2011). Furthermore, Gessi et al. (2013) found that a point mutation in *GNAQ* may be related to the development of primary melanocytic tumors in humans. They identified six cases harboring mutations in codon 209 of the *GNAQ* gene. As members of a cascade of regulatory genes in the melanogenesis pathway, *GNAQ*, *AC*, and *PKA* control the process of melanin production by regulating the expression of upstream or downstream genes (Bennett & Lamoreux, 2003). This suggests that the above‐mentioned genes, which are downregulated in cavefish species, are likely to be closely related to the loss of pigmentation in *Sinocyclocheilus* cavefish.

In addition to the melanogenesis pathway, genes (*Tyr*, *TYRP1*, *P38*, etc.) in Wnt signaling pathway (Fujimura et al., 2009), MAPK signaling pathway and apoptosis pathway (Squarzoni et al., 2011), have been indicated to be involved in the regulation of pigmentation. Tyrosinase, encoded by the *Tyr* gene, is the rate‐limiting enzyme in melanogenesis and is an important regulatory factor in the synthesis of melanin. Previous studies reported that loss or downregulation of *Tyr* gene leads to an albino phenotype in zebrafish (Page‐McCaw et al., 2004), duck (Li et al., 2012), mouse (Ito & Wakamatsu, 2011) and rabbit (Song et al., 2017). Yang et al. (2016) carried out comparative transcriptomic analyses of three *Sinocyclocheilus* fish species, *S. graham* (surface fish), *S. rhinocerous* (cavefish), and *S. anshuiensis* (cavefish) and suggested that *Tyr*, *Tyrp1*, and *Mpv17* (mitochondrial inner membrane protein) lead to the loss of iridophores in *Sinocyclocheilus* cavefish (Yang et al., 2016). In the present study, we did not find significant differences in the expression of *Tyr* or *Tyrp1*. However, we observed that *p38* (mitogen‐activated protein kinase 14) was significantly downregulated in cavefish species compared to the surface fish. In the MAPK signaling pathway, p38 signaling is activated by α‐MSH and UV radiation, thereby promoting the phosphorylation of the *USF‐1* (MITF‐like transcription factor) to activate the *Tyr* promoter (Bellei et al., 2010; Galibert et al., 2001). Furthermore, in this study, some key pigment‐related genes identified in other *Sinocyclocheilus* fish species, such as *Tyr*, *Tyr*, and *Oca2* (Yang et al., 2016), did not show significant differential expression in all cavefish groups. Interestingly, some genes associated with the Wnt, MAPK, and apoptosis pathway, such as *DAAM1* (disheveled associated activator of morphogenesis) and *BID* (BH3 interacting domain death agonist) were significantly downregulated in the skin of most *Sinocyclocheilus* cavefish species studied. This may be partially explained by the spatiotemporal specificity of the transcriptome, or by the fact that these genes show pleiotropy while participating in skin pigment formation. Furthermore, a number of factors could affect the expression of genes in the skin, such as season and individual developmental status. In summary, 11 of DEGs, including *GNAQ*, *PKA*, *NRAS*, and *p38* etc, which are involved in key pigment regulation pathways, were identified through comparative transcriptomics. We infer that these genes may participate in the partial regulation of skin pigmentation, and the downregulation of these genes may lead to the pigmentation loss in *Sinocyclocheilus* cavefish species. Furthermore, we found that the trend of gene expression was different in each group (Table 2), and some genes even no significant difference in some group may due to different mechanisms of pigment loss, which could be responsible for these different species. Therefore, future studies should include gene knockout or overexpression studies to validate the function of these genes.

In this study, we found that the results of qPCR of some pairs may not perfectly support RNA‐seq but they used the same RNA. In order to minimize RNA degradation, in this study, we sent the skin tissue to the sequencing company directly to extract the RNA, and then sent the RNA back to our laboratory to conduct qPCR, we think this may cause the degradation of a small amount of RNA during the sample delivery. Furthermore, since the two experiments are not conduct at the same laboratory, the final results may have some differences.

In conclusion, to further elucidate the underlying genetic mechanism of skin pigment loss in *Sinocyclocheilus* cavefish species, we conducted a comparative histological, WGCNA, and comparative transcriptomics analyses of the skin of *Sinocyclocheilus* fishes dwelling on surface and in caves, and found differences in the distribution, quantity, and morphology of skin melanocytes. A total of 35,101 DEGs were found in four surface‐cave groups. Through GO, COG, and KEGG pathway annotations, we identified 11 candidate genes which showed significant differential expression in *Sinocyclocheilus* cave and surface fish species analyzed in this study, that may participate in the regulation of skin pigmentation. Most of the DEGs were downregulated in *Sinocyclocheilus* cavefishes as validated by qPCR. However, these genes require further functional validation in *Sinocyclocheilus*. This study provides a strong foundation for better understanding of the molecular genetic mechanisms underlying troglomorphic traits in cavefish species.

## CONFLICT OF INTEREST

The authors declare that they have no competing interests.

## AUTHOR CONTRIBUTION

**Chunqing Li:** Formal analysis (lead); Investigation (lead); Methodology (lead). **Hongyu Chen:** Formal analysis (lead); Investigation (lead); Methodology (lead); Writing‐original draft (lead); Writing‐review & editing (lead). **Yinchen Zhao:** Methodology (supporting); Resources (supporting). **Shanyuan Chen:** Conceptualization (lead); Funding acquisition (lead); Project administration (lead); Resources (equal); Supervision (equal); Writing‐original draft (equal); Writing‐review & editing (equal). **Heng Xiao:** Conceptualization (lead); Funding acquisition (lead); Project administration (equal); Resources (equal); Supervision (equal).

## ETHICS APPROVAL AND CONSENT TO PARTICIPATE

The *Sinocyclocheilus* fish species used in this study were caught from wild water bodies, and no specific permissions were required. All experiments were conducted after review and approval from the local Ethical Committee at Yunnan University in accordance with China's local and global ethical policies (Grant No: ynucae 20190056), and all the procedures were approved and assisted by the local government.

## CONSENT FOR PUBLICATION

Not applicable.

## Supporting information

Fig S1

Fig S2

Fig S3

Fig S4

Fig S5

Table S1‐S6

## Data Availability

All data generated or analyzed during this study are included in this published article and its supplementary information files. The raw reads produced in this study were deposited in the DRYAD database (https://datadryad.org/stash/share/t5cZIXoVUgyhpzEP6z-GN6xjc5EU3TvPPEwbdIo7siI).
