# Supplementary figures and images for: Comparative transcriptomics reveals the molecular genetic basis of pigmentation loss in Sinocyclocheilus cavefishes
Source: Ecol Evol. 2020 Nov 19;10(24):14256–71. doi: 10.1002/ece3.7024 (PMC7771137; doi:10.1002/ece3.7024)

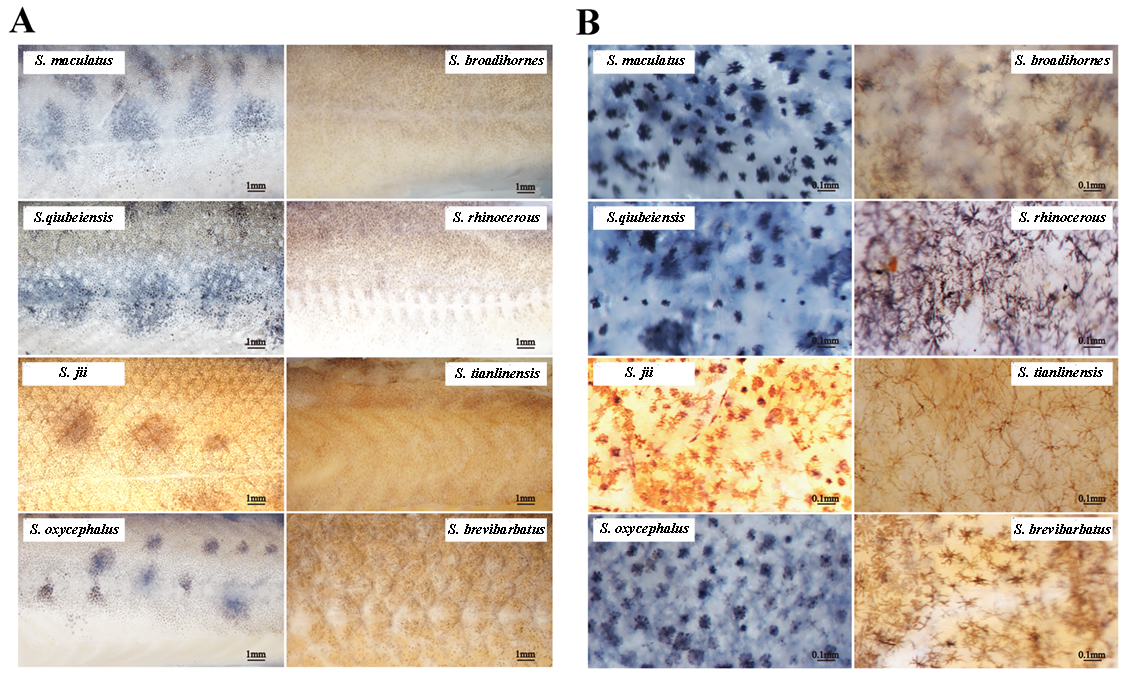

Supplement: Supplementary file 1 — Fig S1 [file ECE3-10-14256-s001.tif]

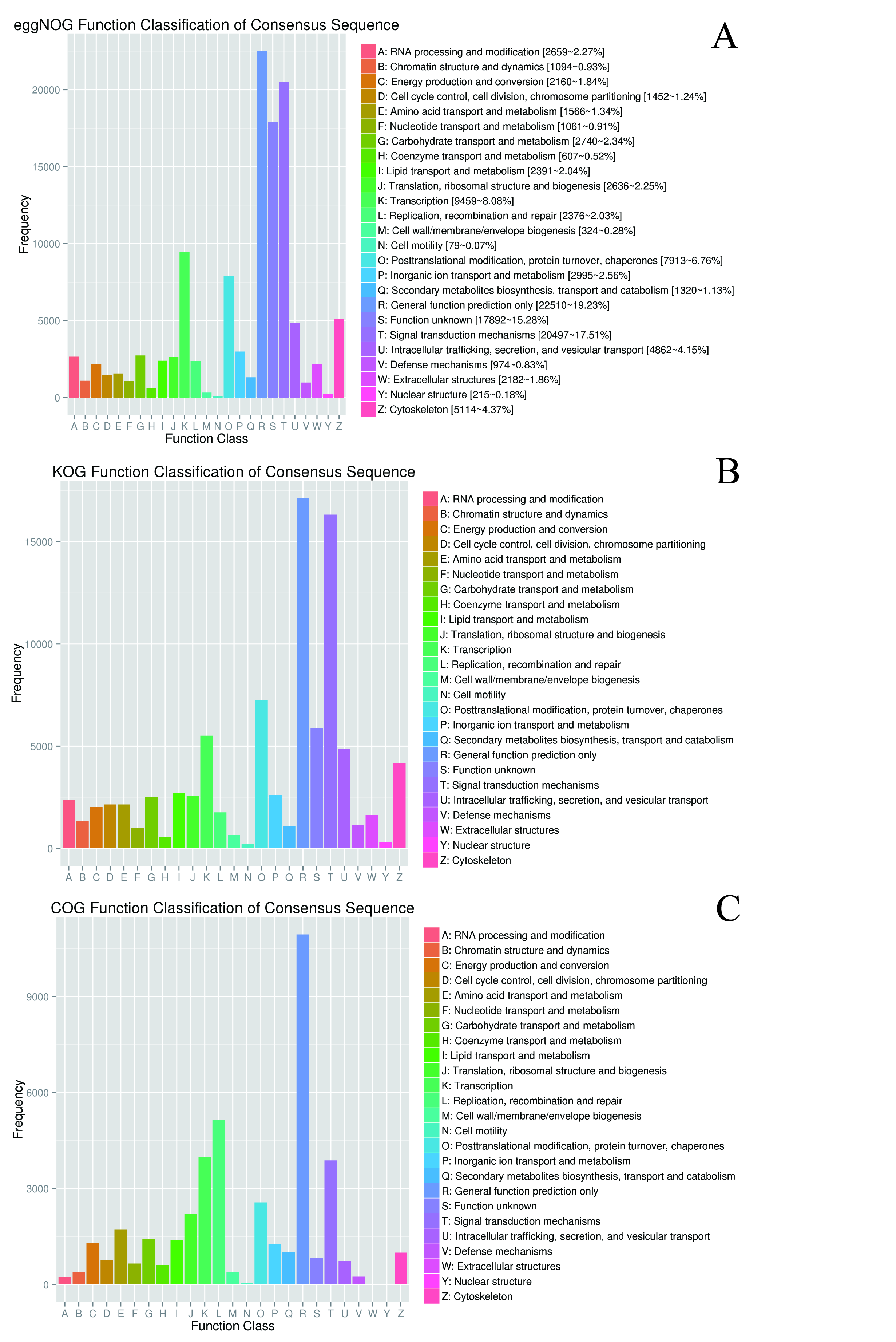

Supplement: Supplementary file 2 — Fig S2 [file ECE3-10-14256-s002.tif]

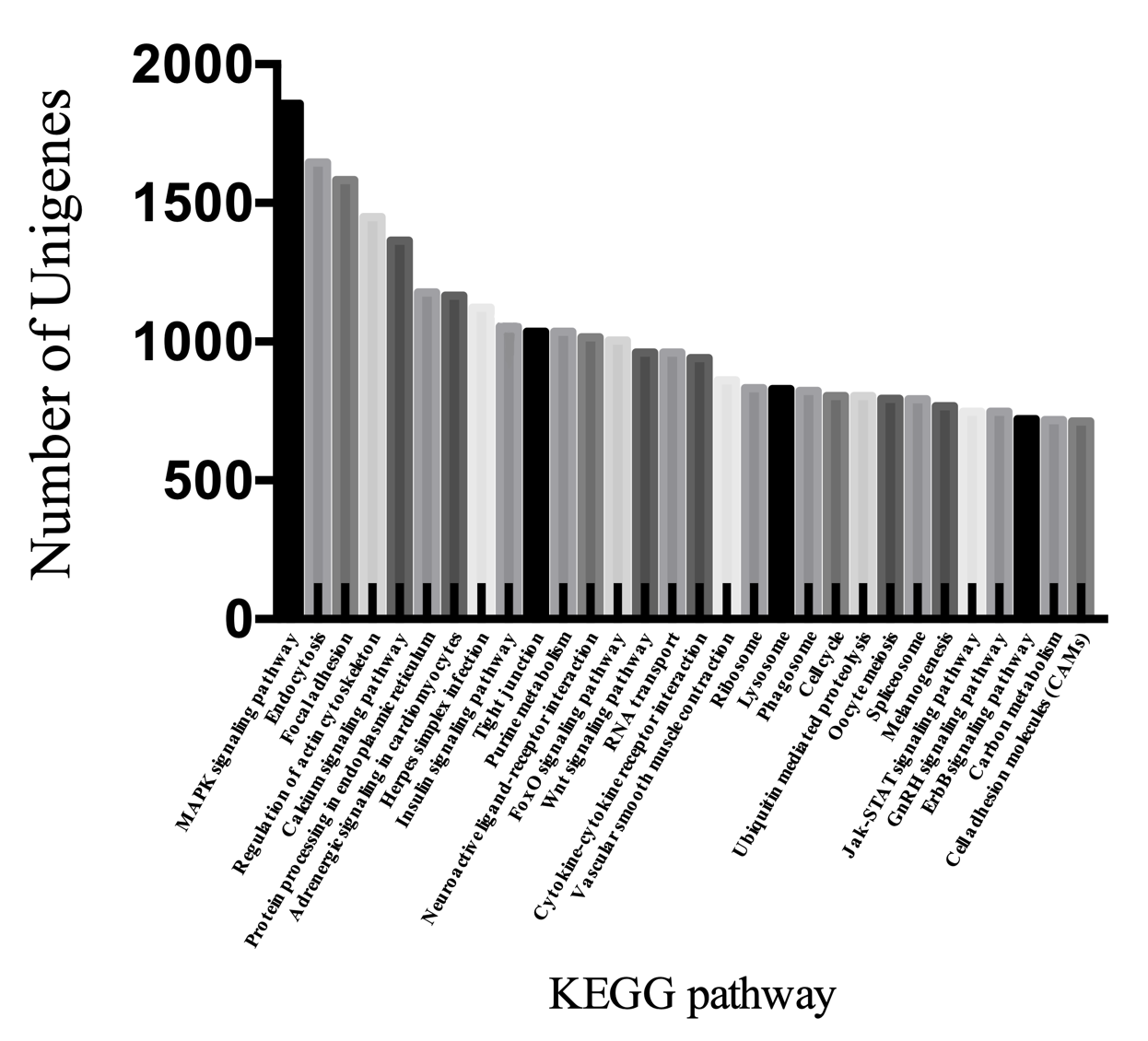

Supplement: Supplementary file 3 — Fig S3 [file ECE3-10-14256-s003.tif]

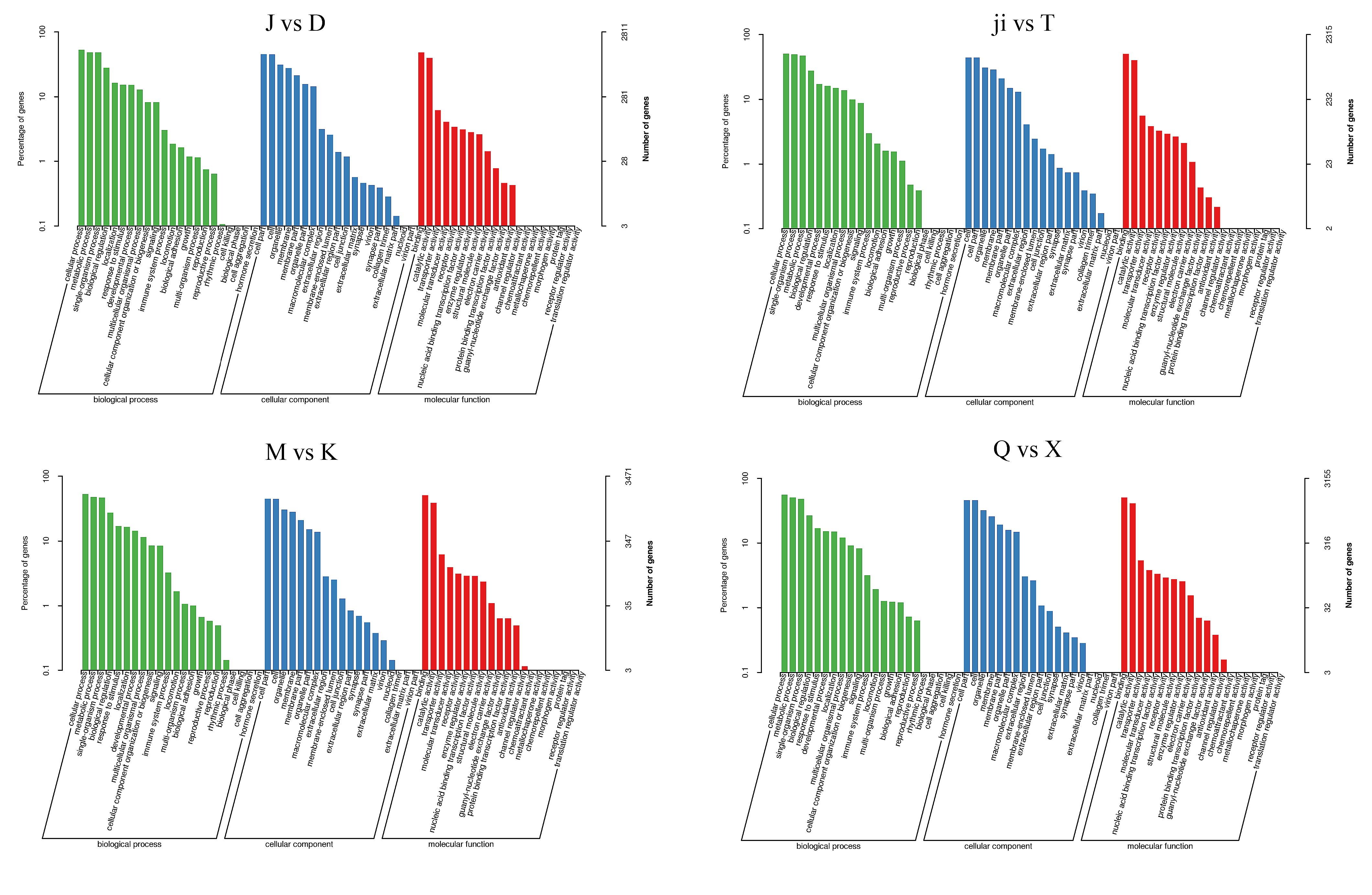

Supplement: Supplementary file 4 — Fig S4 [file ECE3-10-14256-s004.tif]

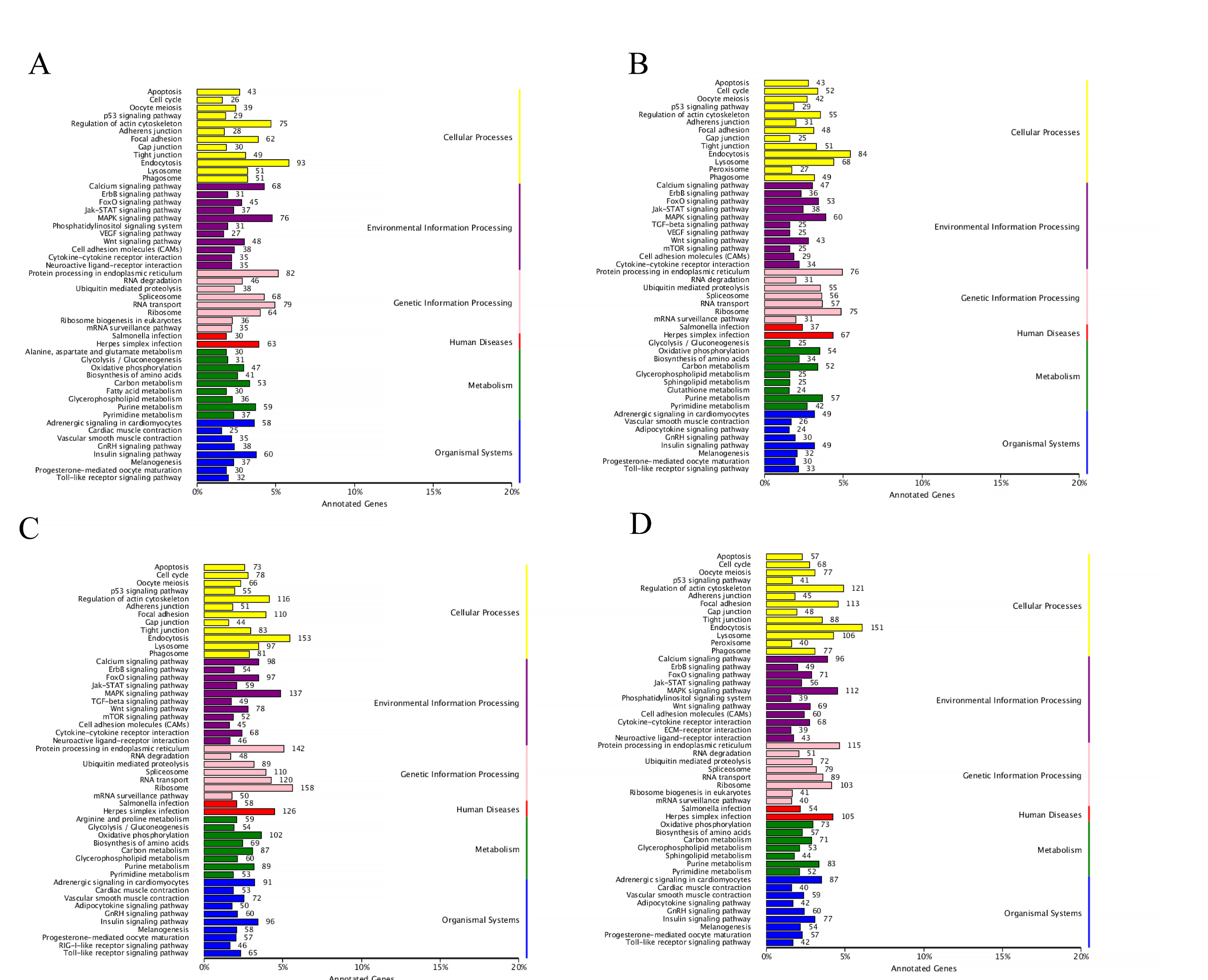

Supplement: Supplementary file 5 — Fig S5 [file ECE3-10-14256-s005.tif]
