# Supplementary material for: Comparative transcriptomics reveals the molecular genetic basis of pigmentation loss in Sinocyclocheilus cavefishes
Source: Ecol Evol. 2020 Nov 19;10(24):14256–71. doi: 10.1002/ece3.7024 (PMC7771137; doi:10.1002/ece3.7024)
Supplement: Supplementary file 6 — Table S1‐S6 [file ECE3-10-14256-s006.docx]

Table 1. Primers used for qRT-PCR

| **gene** | **Primer F** | **Primer R** | **TM** |
| --- | --- | --- | --- |
| TAO | GGAGAGACGAGAGATGTA | ATGAACTGGCTGAGAATG | 57.5 |
| P38 | TCTTGACCTTCTCTTCTGGAT | CCTACTGATTGGCTGATTACAA | 55.9 |
| AC | AAGCAGGAAGGAAGTAGAG | AAGAAGACGAGGCAGATG | 58.4 |
| NRAS | GCCATAAACAACAGCAAATC | GAACATCATCGGAGTCCTT | 56.4 |
| DAAM1 | CTGATGTCACGCCATTAGA | CCTGTTCCTTCCACTGTT | 54.9 |
| SFRP2 | ACTATGAGAACAGGTTGGTA | TGAGAGGAGACAGAACAC | 55.5 |
| GNAQ | GCTTCCTCATTGATGTCT | TCTCTGTCTATTGCTCCAT | 55.0 |
| BID | CAGAGACAGAGAAGACAGA | TGAGCCACATAGATGAGT | 54.2 |
| PKA | GTAACAAGTGACGAAGGAT | GGTGATGATGATGATGATGA | 59.0 |
| β-Actin | GACCACCTTCAACTCCAT | ACCACCAGACAATACAGT | 56.5 |

| **Samples** | **ID** | **Clean Read Number** | **Base Number** | **GC Content** | **%≥Q30** |
| --- | --- | --- | --- | --- | --- |
| M | T01 | 54,823,753 | 16,240,657,444 | 48.02% | 92.19% |
|  | T02 | 29,074,237 | 8,611,275,452 | 47.06% | 91.79% |
|  | T03 | 56,429,950 | 16,754,567,358 | 47.45% | 92.12% |
| Q | T04 | 35,508,709 | 10,527,068,386 | 48.20% | 91.61% |
|  | T05 | 22,969,435 | 6,808,518,802 | 47.88% | 91.30% |
|  | T06 | 25,530,549 | 7,568,677,822 | 47.82% | 91.53% |
| ji | T07 | 47,942,374 | 14,204,680,558 | 48.25% | 92.03% |
|  | T08 | 42,120,505 | 12,461,397,088 | 47.64% | 92.01% |
|  | T09 | 41,593,369 | 12,292,941,008 | 48.81% | 92.49% |
| T | T10 | 23,921,852 | 7,089,947,010 | 46.36% | 92.06% |
|  | T11 | 28,586,647 | 8,467,832,880 | 48.10% | 91.73% |
|  | T12 | 37,785,958 | 11,210,566,926 | 47.72% | 91.81% |
| D | T13 | 32,033,650 | 9,500,523,020 | 48.42% | 91.28% |
|  | T14 | 36,944,123 | 10,960,652,020 | 48.09% | 91.49% |
|  | T15 | 22,296,068 | 6,613,328,490 | 48.71% | 91.16% |
| K | T16 | 34,262,325 | 10,152,443,698 | 47.23% | 91.42% |
|  | T17 | 27,625,831 | 8,201,843,168 | 46.87% | 90.80% |
|  | T18 | 28,508,450 | 8,465,134,518 | 46.15% | 91.20% |
| J | T19 | 30,659,982 | 9,087,855,802 | 47.60% | 91.16% |
|  | T20 | 27,793,849 | 8,240,196,706 | 47.31% | 91.43% |
|  | T21 | 31,707,601 | 9,404,131,824 | 47.25% | 91.64% |
| X | T22 | 27,161,319 | 8,046,703,190 | 47.87% | 91.30% |
|  | T23 | 30,345,437 | 8,993,507,046 | 47.44% | 91.30% |
|  | T24 | 27,172,934 | 8,041,706,300 | 47.07% | 91.26% |
| SUM |  | 802,798,907 | 237,946,156,516 |  |  |

Table 2. Sequencing data for eight *Sinocyclocheilus* species

| **Length Range** | **All Unigenes** | **M Unigenes** | **Q Unigenes** | **ji Unigenes** | **T Unigenes** | **D Unigenes** | **K Unigenes** | **J Unigenes** | X **Unigenes** |
| --- | --- | --- | --- | --- | --- | --- | --- | --- | --- |
| 200-300 | 62,399(20.94%) | 31,487(33.21%) | 30,340(33.74%) | 19,752(27.38%) | 36,478(34.18%) | 29,929(34.09%) | 31,706(35.81%) | 38,302(36.72%) | 31,738(33.37%) |
| 300-500 | 47,245(15.85%) | 21,901(23.10%) | 20,670(22.98%) | 14,883(20.63%) | 24,685(23.13%) | 19,878(22.64%) | 20,879(23.58%) | 24,888(23.86%) | 21,513(22.62%) |
| 500-1000 | 73,504(24.67%) | 19,932(21.02%) | 18,565(20.64%) | 16,763(23.24%) | 23,034(21.59%) | 17,424(19.85%) | 16,971(19.17%) | 20,457(19.61%) | 19,358(20.35%) |
| 1000-2000 | 65,958(22.13%) | 13,276(14.00%) | 12,795(14.23%) | 13,329(18.48%) | 15,534(14.56%) | 12,859(14.65%) | 11,616(13.12%) | 13,855(13.28%) | 14,325(15.06%) |
| 2000+ | 48,874(16.40%) | 8,226(8.68%) | 7,560(8.41%) | 7,417(10.28%) | 6,977(6.54%) | 7,703(8.77%) | 7,359(8.31%) | 6,805(6.52%) | 8,178(8.60%) |
| Total Number | 297,984 | 94,822 | 89,931 | 72,145 | 106,709 | 87,793 | 88,531 | 104,307 | 95,112 |
| Total Length | 340,964,006 | 74,716,389 | 70,021,665 | 64,468,043 | 77,471,546 | 69,653,609 | 67,324,345 | 73,831,239 | 75,147,480 |
| N50 Length | 1,881 | 1,337 | 1,325 | 1,468 | 1,159 | 1,387 | 1,335 | 1,165 | 1,356 |
| Mean Length | 1144.24 | 787.96 | 778.62 | 893.59 | 726.01 | 793.38 | 760.46 | 707.83 | 790.09 |

Table 3. Summary of unigenes identified in skin samples of the eight *Sinocyclocheilus* species

Table 4. Gene annotation in different databases

| **Anno_Database** | **Annotated_Number** | **300<=length<1000** | **length>=1000** |
| --- | --- | --- | --- |
| COG_Annotation | 30146 | 5378 | 23338 |
| GO_Annotation | 67419 | 14857 | 49971 |
| KEGG_Annotation | 60928 | 13933 | 44105 |
| KOG_Annotation | 79871 | 17100 | 58778 |
| Pfam_Annotation | 89484 | 17872 | 68211 |
| Swissprot_Annotation | 68653 | 13999 | 52388 |
| eggNOG_Annotation | 117078 | 30913 | 78273 |
| nr_Annotation | 126654 | 33918 | 85691 |
| All_Annotated | 131766 | 36137 | 86532 |

Table 5. Summary of differentially expressed genes (DEGs) and Annotation in different databases

|  | **All_DEG** | **up-regulated** | **down-regulated** | **COG** | **GO** | **KEGG** | **eggNOG** | **nr** |
| --- | --- | --- | --- | --- | --- | --- | --- | --- |
| M vs K | 10629 | 4332 | 6297 | 1715 | 3471 | 2903 | 5566 | 6260 |
| Q vs X | 9635 | 3628 | 6007 | 1633 | 3155 | 2793 | 5072 | 5463 |
| ji vs T | 8395 | 2604 | 5791 | 744 | 1525 | 1647 | 2300 | 2433 |
| J vs D | 6442 | 2976 | 3466 | 1005 | 2315 | 1913 | 3606 | 3980 |

| Table 6. The extremely significant (P<0.001) GO terms of differentially expressed genes in the De novo transcriptome analysis | | |
| --- | --- | --- |
|  | Category | Term (GO.ID) |
| M_vs_K | BP | homophilic cell adhesion via plasma membrane adhesion molecules (GO:0007156) |
|  | BP | ionotropic glutamate receptor signaling pathway (GO:0035235) |
|  | BP | potassium ion transmembrane transport (GO:0071805) |
|  | BP | sodium ion transmembrane transport (GO:0035725) |
|  | CC | extracellular region (GO:0005576) |
|  | CC | ribosome (GO:0005840) |
|  | CC | plasma membrane (GO:0005886) |
|  | CC | cation channel complex (GO:0034703) |
|  | CC | plasma membrane part (GO:0044459) |
|  | CC | integral component of membrane (GO:0016021) |
|  | MF | structural constituent of ribosome (GO:0003735) |
|  | MF | ionotropic glutamate receptor activity (GO:0004970) |
|  | MF | extracellular-glutamate-gated ion channel activity (GO:0005234) |
| QB_vs_XJ | BP | homophilic cell adhesion via plasma membrane adhesion molecules (GO:0007156) |
|  | BP | nervous system development (GO:0007399) |
|  | BP | regulation of ion transmembrane transport (GO:0034765) |
|  | BP | ionotropic glutamate receptor signaling pathway (GO:0035235) |
|  | CC | extracellular region (GO:0005576) |
|  | CC | plasma membrane part (GO:0044459) |
|  | CC | ribosomal subunit (GO:0044391) |
|  | MF | structural constituent of ribosome (GO:0003735) |
|  | MF | anion:cation symporter activity (GO:0015296) |
|  | MF | ionotropic glutamate receptor activity (GO:0004970) |
|  | MF | extracellular-glutamate-gated ion channel activity (GO:0005234) |
| JS_vs_TL | BP | phospholipid biosynthetic process (GO:0008654) |
|  | BP | alternative mRNA splicing, via spliceosome (GO:0000380) |
|  | BP | somitogenesis (GO:0001756) |
|  | BP | chromatin modification (GO:0016569) |
|  | BP | DNA damage response, signal transduction by p53 class mediator resulting in transcription of p21 class mediator (GO:0006978) |
|  | BP | peripheral nervous system neuron axonogenesis (GO:0048936) |
|  | CC | cell-cell junction (GO:0005911) |
|  | CC | intracellular organelle part (GO:0044446) |
|  | CC | nuclear envelope (GO:0005635) |
|  | CC | polysome (GO:0005844) |
|  | MF | cation-transporting ATPase activity (GO:0019829) |
|  | MF | sphingomyelin phosphodiesterase activity (GO:0004767) |
|  | MF | interleukin-1 receptor binding (GO:0005149) |
|  | MF | GTPase activity (GO:0003924) |
|  | MF | kinase activity (GO:0016301) |
| JT_vs_DX | BP | homophilic cell adhesion via plasma membrane adhesion molecules (GO:0007156) |
|  | BP | ionotropic glutamate receptor signaling pathway (GO:0035235) |
|  | BP | monocarboxylic acid transport (GO:0015718) |
|  | BP | muscle structure development (GO:0061061) |
|  | BP | negative regulation of T cell activation (GO:0050868) |
|  | CC | plasma membrane part (GO:0044459) |
|  | CC | extracellular region (GO:0005576) |
|  | CC | cell junction (GO:0030054) |
|  | CC | ribosome (GO:0005840) |
|  | CC | plasma membrane (GO:0005886) |
|  | MF | structural constituent of ribosome (GO:0003735) |
|  | MF | ionotropic glutamate receptor activity (GO:0004970) |
|  | MF | extracellular-glutamate-gated ion channel activity (GO:0005234) |
|  | MF | glycogenin glucosyltransferase activity (GO:0008466) |
|  | MF | calcium ion binding (GO:0005509) |
